# Studies on Pathological Changes in the Gastrointestinal Tract Due to Helminth Infection in Backyard Chickens

**DOI:** 10.1155/japr/6446780

**Published:** 2026-07-03

**Authors:** Sahar Bhutto, Sadaf Khushk, Mansoor Tariq, Javaid Ali Gadahi, Sana Noor Panhwer, Zaheer Ahmed Nizamani, Shaharbano Memon

**Affiliations:** ^1^ Department of Veterinary Pathology, Sindh Agriculture University, Tandojam, Pakistan, sau.edu.pk; ^2^ Department of Veterinary Parasitology, Sindh Agriculture University, Tandojam, Pakistan, sau.edu.pk; ^3^ Department of Veterinary Parasitology, Shaheed Benazir Bhutto University of Veterinary and Animal Sciences, Sakrand, Sindh, Pakistan, sbbuvas.edu.pk; ^4^ Department of Animal Breeding and Genetics, Sindh Agriculture University, Tandojam, Pakistan, sau.edu.pk

**Keywords:** *A*. *sphenoides*, backyard chicken, helminthiasis, indigenous chicken, Pakistan

## Abstract

Backyard poultry farming is an important aspect for enhancing Pakistan′s rural economy, yet helminth infections remain a major issue for flock health and productivity. This study was conducted to assess the prevalence of gastrointestinal helminth parasites and to record the associated gross and histopathological lesions in backyard chickens from 12 villages in and around Tandojam City, Sindh, Pakistan, between August and December 2022. A total of 76 adult chickens of both sexes were selected through systematic random sampling. Each chicken underwent a complete necropsy, and the gastrointestinal tract was carefully examined for helminth infestation. Tissue samples were collected and processed for histopathological evaluation using hematoxylin and eosin (H&E) staining. Out of 76 chickens examined, 68 (89.47%) were infected with parasites. Four species of cestode were isolated: *Cotugnia digonopora* (*C. digonopora*) (32.89%), *Choanotaenia infundibulum* (*C. infundibulum*) (27.63%), *Raillietina cesticillus* (*R. cesticillus*) (14.47%), and *Amoebotaenia sphenoides* (*A. sphenoides*) (3.94%). In addition, two nematodes were identified: *Heterakis gallinarum* (*H. gallinarum*) (43.42%) and *Ascaridia galli* (*A. galli*) (35.53%). Mixed infections involving both *H. gallinarum* and *A. galli* occurred in 12 birds (15.79%). Gross pathological changes included intestinal obstruction with bloody exudates, ballooning and enlargement of the ceca, petechial hemorrhages in the duodenum, and mild hepatic inflammation. Histopathologically, all infected chickens showed inflammatory cell infiltration, mucosal erosion, villous degeneration, and hemorrhagic enteritis. Notably, scolices of *R. cesticillus* and *A. sphenoides* were embedded within the intestinal epithelium. The detection of *A. sphenoides* in backyard chickens represents the first confirmed record of this species in Sindh Province, Pakistan. These findings highlight the high burdens of helminth infections in rural poultry and underscore the urgent need for routine anthelmintic treatment and strengthened biosecurity measures in backyard poultry across Sindh.

## 1. Introduction

Pakistan′s poultry industry is a vital part of the agriculture sector, contributing 1.3% to the national GDP and experiencing an annual growth rate of 8%–10% [1, 2]. Additionally, backyard poultry farming is also important for supporting rural farmers. Traditionally, in rural areas, people have raised native poultry breeds, such as Aseel and Desi, along with exotic breeds like Black Australorp, Rhode Island Red, Naked Neck, and hybrid Fayoumi, for meat, eggs, or both to sustain their livelihoods [3]. Backyard poultry farming provides subsistence income and high‐quality, valuable food that enhances household nutrition [4]. Moreover, it is usually raised under free‐range, semi‐intensive, or intensive conditions, with feeding practices adapted to available feed resources. Furthermore, backyard chickens play an important role by providing easy access to stress‐free, environmentally safe birds [5–6]. However, backyard farming remains the fourth lowest prioritized sector of poultry farming in terms of biosecurity [7]. Because of poor preventive and management factors, poultry birds are prone to infections and pose a zoonotic threat to humans [8].

Parasitic infections in backyard poultry cause serious consequences for their health, leading to reduced productivity and increased mortality rates. These infections primarily involve roundworms (nematodes), tapeworms (cestodes), and flukes (trematodes), which are recognized as major contributors to helminthiasis [9–10]. Additionally, the most reported helminth species in chicken populations were *A. galli* (35.9%), *H. gallinarum* (28.5%), *Capillaria* spp. (5.9%), and *Raillietina* spp. (19.0%) [11]. However, the significant occurrence of roundworm in chicks usually begins at a young age [12]. Infected birds shed worms and eggs in feces that are ingested by intermediate hosts, including beetles, cockroaches, earthworms, flies, and grasshoppers [13]. Common gastrointestinal nematodes in backyard chickens cause decreased egg production, diarrhea, intestinal obstruction, morbidity, significant hemoglobin depression, and increased mortality [14]. *A. galli* is the largest gut‐dwelling helminth affecting chickens. Both adult and immature stages of this parasite impact gut health, but the larval forms play a significant role in causing pathology. The parasite has been linked to anemia, weight loss, and decreased production efficiency [15]. In severe infections, *A. galli* can obstruct the small intestine, leading to marked inflammation, increased mucus production, and petechial hemorrhages, particularly in the duodenum [16]. Moreover, *H. gallinarum*, another of the most common roundworms, infects a wide range of birds [17]. It causes severe damage to the cecal architecture, lamina propria necrosis, and destruction of the intestinal glands. The infected cecum showed muscular alterations, including vacuolation and muscle tearing [18].

Cestodes are also considered major gastrointestinal parasites of backyard chickens [19]. Climatic changes, agroecological zones, and the populations of intermediate hosts, such as beetles and house flies that harbor cestode larvae or eggs, may affect the prevalence in susceptible hosts [20, 21]. These parasites cause decreased egg production, diarrhea, intestinal blockage, substantial hemoglobin drop, morbidity, and a high mortality rate in backyard chickens [14, 20]. Free‐range chickens ingest dung beetles and ants that carry the *cestode Raillietina* spp., which accumulate in the jejunum and ileum of primary hosts and cause reduced growth, emaciation, weakness, and obstruction of the digestive tract [22], their larval stage (cysticercoid) lives in a variety of invertebrate intermediate hosts, including ants, beetles, mini‐wasps, and termites [23, 24]. Moreover, *Dermestes maculatus* DeGeer (beetle) serves as a vector of *R. cesticillus*, which also causes damage to the intestines. Histopathologically, gut‐associated lymphoid tissues were observed in the jejunum lamina propria, which led to a decline in egg production [25]. *R. echinobothridia* produces nodules that may be confused with avian tuberculosis lesions [26].

Histopathological finding of *C. infundibulum* revealed severe intestinal damage, including distortion, fusion, inflammation, and destruction of the architecture of muscular layers, villi, and glands [27]. *A. sphenoides* infection is also reported in backyard chickens [28], causing intestinal irritation and reducing productivity.

The present study was conducted to evaluate the prevalence of helminth infections and to examine the pathological and histopathological lesions in the gastrointestinal tract (GIT) of backyard poultry. Most parasites in domestic chickens can be harmful, though their effects vary depending on the habitat and the availability of clean water and food, which can differ by region. Additionally, helminth infections lead to significant production losses in backyard poultry. Limited treatment options and the difficulty of controlling intermediate hosts present challenges for farmers. These factors result in economic losses in backyard farming, characterized by delayed growth, low weight gain, and decreased productivity [29].

## 2. Materials and Methods

### 2.1. Ethical Considerations

This study involves the humane collection of chickens′ gastrointestinal tracts. The method of euthanasia was carefully evaluated, and we obtained ethical clearance from the Sindh Agriculture University Ethics Review Committee (SAUERC), Reference Number PATH/23/2022/12. All procedures related to the slaughter of live birds were performed in strict accordance with the institutional guidelines.

### 2.2. Experimental Design

The present study was carried out on a total of 76 (n = 76) adult backyard chickens of both sexes. The sample size was determined based on the estimated helminth prevalence reported in previous studies [30]. To conduct our study, we implemented a systematic random sampling method across 12 villages in and around Tandojam City, Sindh. This area is known for its dense poultry farming background and a lack of accessible veterinary services. Within each village, every third household keeping chickens was approached, and one adult bird per household was included. Only birds exhibiting clinical signs of helminthiasis (including reduced feed intake, weight loss, diarrhea, or general debilitation) were selected for necropsy examination. Sampling was conducted from August to December 2022, spanning the late‐summer‐to‐early‐winter transition. Birds were brought alive to the Department of Veterinary Pathology, Faculty of Animal Husbandry and Veterinary Sciences, Sindh Agriculture University, Tandojam, for further examination.

### 2.3. Gross Inspection of the Digestive Tract for the Presence of Helminth Parasites

Birds were slaughtered following ethical consideration and the halal slaughtering method. Afterward, a complete necropsy was performed on each bird. The abdominal cavity was opened by a midline incision through the peritoneum using a scalpel, and the entire GIT was removed and placed in a labeled petri dish. The lumen of each gastrointestinal segment was opened by longitudinal incision and examined for the presence and identification of helminth parasites visible to the naked eye. All parasites were collected with tissue forceps, counted, and washed in normal saline. Washed parasites were preserved in a glass bottle containing 10% formalin following the procedure described by [31]. The intensity of gross pathological lesions caused by these parasites was graded using a 4‐point scoring system (Table 1) as described in [32]. All morbid tissues (intestine and ceca) were collected in 10% neutral formalin for histopathological processing.

**Table 1:** Scoring criteria for gross pathological lesions.

Score	Remarks
Score 0 (−)	No lesions observed
Score 1 (+)	Mild lesions observed
Score 2 (++)	Moderate lesions observed
Score 3 (+++)	Severe lesions observed

### 2.4. Histopathology of the Affected Intestine

The intestinal and cecal samples were collected and preserved in 10% neutral buffer formalin for histopathological examination using the standard paraffin impregnation technique. Tissue samples were first washed under running distilled water for 2 h to remove residual formalin. Dehydration was carried out by passing samples sequentially through graded ethanol concentrations of 60%, 70%, 90%, and 100% (I) and 100% (II), with each step maintained for 2 h. Samples were then cleared in Xylene I and II for 2 h each. For infiltration, paraffin wax was heated in a hot‐air oven at 60°C for 6 h, and tissue blocks were prepared in metal molds. Sections of 4‐ to 5‐*μ*m thickness were cut using a rotary microtome. Tissue ribbons were floated in a gelatinized water bath at 37°C for 45–60 s, mounted on microscope slides, and stained with hematoxylin and eosin (H&E) stain. Stained slides were examined under a light microscope for the pathological lesions associated with helminth parasites.

### 2.5. Staining Protocol and Identification of Helminth Parasites

Recovered worms were dorsoventrally compressed between two glass slides, fixed in 10% formalin, and dehydrated through ascending grades of ethyl alcohol (40%, 60%, 80%, and 100%). Helminth parasites were stained with Field stain, cleared in xylene, and then mounted in Canada balsam on clean glass slides with coverslips. Scolexes were gently compressed between two coverslips to maintain the intact outline of suckers, rostella, and hooks. Prepared slides were carefully examined under a light microscope, and recovered cestodes were identified according to [27].

### 2.6. Statistical Analysis

All collected data were analyzed in this study using Microsoft Excel (Version 2019). Descriptive statistics were applied to determine the frequency and percentage prevalence of helminth species. Parasite prevalence was expressed as the percentage of birds positive for a given species out of the total birds examined. Relative density (abundance) was calculated as the total number of parasites of a given species recovered divided by the total number of hosts examined, including both infected and uninfected birds.

Relative density or abundance=Total number of parasitesTotal number of hosts examined

## 3. Results

### 3.1. Parasite Identification

In the present study, 68 of 76 chickens (89.47%) were positive for at least one helminth species, whereas eight (10.52%) were negative. Sixty out of 76 birds (78.94%) were positive for cestode infection, and eight (10.52%) showed mixed cestode and nematode infections. Cestode infections were confirmed by gross inspection and further confirmed by microscopic examination, which revealed *C. digonopora*, *C. infundibulum*, *R. cesticillus*, and *A. sphenoides* infection in backyard poultry (Figures 1 and 2). Out of 76 birds, 25 (32.89%) were positive for *C*. *digonopora*, 21 (27.63%) for *C. infundibulum*, 11 (14.47%) for *R. cesticillus*, and 3 (3.94%) for *A. sphenoides*.

**Figure 1:**
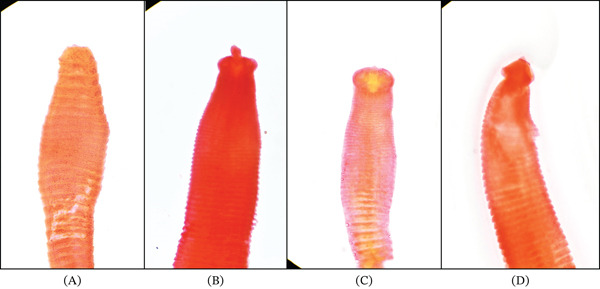
Microscopic identification of cestode species revealed from backyard chicken. The scolex of the (A) *A. sphenoides*, (B) *C. infundibulum*, (C) *C. digonopora*, and (D) *Raillietinna* spp.

**Figure 2:**
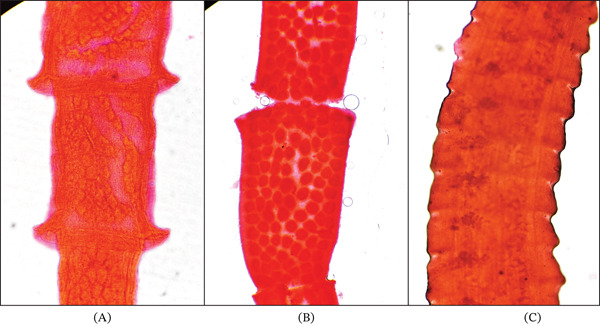
Microscopic identification of cestode species with parts of the body. The gravid proglottid of the (A) *C. infundibulum*, (B) *R. cesticillus*, and (C) *C. digonopora*.

Roundworm infection: 60 out of 76 chickens (78.94%) were positive for nematode infection. All positive cases were confirmed through microscopic examination (Figure 3). Two nematode species were identified: *H. gallinarum* and *A. galli*. Thirty‐three out of 76 chickens (43.42%) were positive for *H. gallinarum*, and 27 out of 76 chickens (35.53%) were positive for *A. galli*. Twelve chickens (15.79%) showed mixed infections with both *H. gallinarum* and *A. galli*.

**Figure 3:**
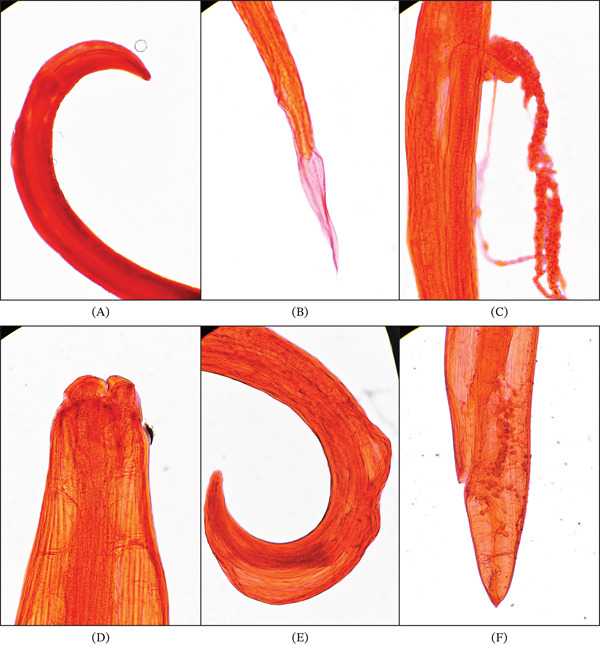
Microscopic identification of nematode species with parts of the body. (A, E) Adult *H. gallinarum*′s front end displays shortened lips, narrowing peripheral alae, and a solid posterior bulb‐shaped esophagus. (B) Adult female *H. gallinarum* with a sharp and tapering posterior end. (C) *H. gallinarum* adult male middle edge showing subequal spicules protruding from the body. (D) Simple club‐shaped esophagus can be seen on the anterior end of adult *A. galli*. (F) Adult female *A. galli* with a conical and straight posterior end. Magnification 400x.

### 3.2. Gross Pathological Findings

Gross pathological lesions were recorded in the intestine and ceca of 68 out of 76 examined backyard chickens (89.47%) (Figure 4). Out of the 68 infected chickens, 42 (61.76%) showed simultaneous infestations of two or more helminth species, with multiple cestode and nematode species recovered from the intestine and ceca. Gross pathological lesions in the intestine and ceca were attributed to four of the cestodes: *C. digonopora*, *C. infundibulum*, *R. cesticillus*, and *A. sphenoides.* The accumulation of large cestodes (12–18 cm long) resulted in partial to complete clogging of the intestinal lumen, accompanied by bloody exudation, and conforming to the moderate to severe pathological lesion score (2+ to 3+) as described in Table 2. The severity of enteric and cecal changes was related to the cestode burden; attachment of cestode scolex caused hemorrhagic lesions and erosion of the intestinal mucosa. All infected backyard chickens were mainly infested with *C. digonopora*, followed in decreasing order of prevalence by *C. infundibulum*, *R. cesticillus*, and *A. sphenoides.* The most heavily parasitized segments were the lower small intestine distal to the duodenum, whereas the fewest cestodes were recovered from the region near the cloaca. Necropsy findings showed that infected birds were anemic, with enteritis, diarrhea, and intestinal hemorrhages consistently observed in heavily infested chickens. Given that nematodes recovered in relatively small numbers per bird, the gross pathological changes were attributed specifically to *H. gallinarum* and *A. galli*, which could not always be distinguished from those caused by concurrent cestode infections. Most adult acaridans were localized in the duodenum and ileum, where the lumen was clogged with mucus and bloody exudate. Petechial hemorrhages were evident in the duodenal mucosa. Small, white nodules measuring approximately 2–3 mm in diameter were detected in the cecal mucosa of birds positive for *H. gallinarum.* Mild to moderate gross pathological lesion scores were assigned to the colon, ceca, and liver individually in each bird during necropsy examination.

**Figure 4:**
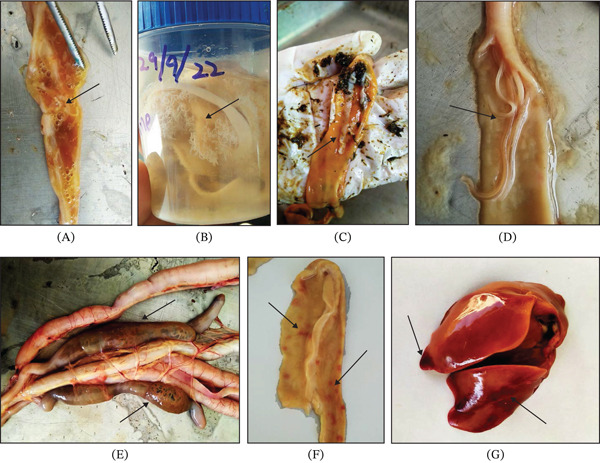
Gross pathological lesions in the intestines of backyard chickens. Cestodes exhibiting (A) a bubble‐like and foggy appearance, (B) a cluster of *Raillietinna* spp. embedded within the chicken′s intestinal epithelium, (C) gross pathological lesions in the intestinal lumen along with the presence of a cestode, (D) occurrence of *A. galli* within the intestinal lumen, (E) ballooning of the ceca, (F) petechial hemorrhages observed in the duodenal region of the small intestine, (G) and inflammation of the liver.

**Table 2:** Severity of gross pathological lesions by organ.

Organ	Remarks	Score
Intestine	Severe pathological lesions in the intestine (enteritis); mucus and bloody exudate in the small and large intestine with frothy, enlarged, yellowish contents.	Severe 3 (+++)
Ceca	Severe pathological lesions in the ceca (typhlitis), enlarged ceca with a ballooning appearance, and cestodes in the ceca of the chicken.	Moderate 2 (++)
Liver	The liver was found inflamed and enlarged.	Mild 1 (+)

### 3.3. Histopathology Findings

Histopathology examination of intestinal tissues from all infected backyard chickens revealed persistent evidence of parasite‐induced mucosal damage. The most frequently observed changes were degeneration and necrosis of intestinal villi and erosion of the mucosal epithelium. Scolices of *R. cesticillus and A. sphenoides* were identified embedded within the intestinal epithelial layer (Figure 5). Moreover, necrosis of the surrounding epithelium and shedding of parasite eggs occurred throughout the intestinal lumen (Figure 6). Hemorrhagic enteritis was associated with severe villous loss and degenerative changes in the intestinal wall. In severely affected birds, a prominent inflammatory cell infiltrate was observed throughout the GIT, accompanied by hyperemia, hemorrhage, and epithelial erosion (Figure 5).

**Figure 5:**
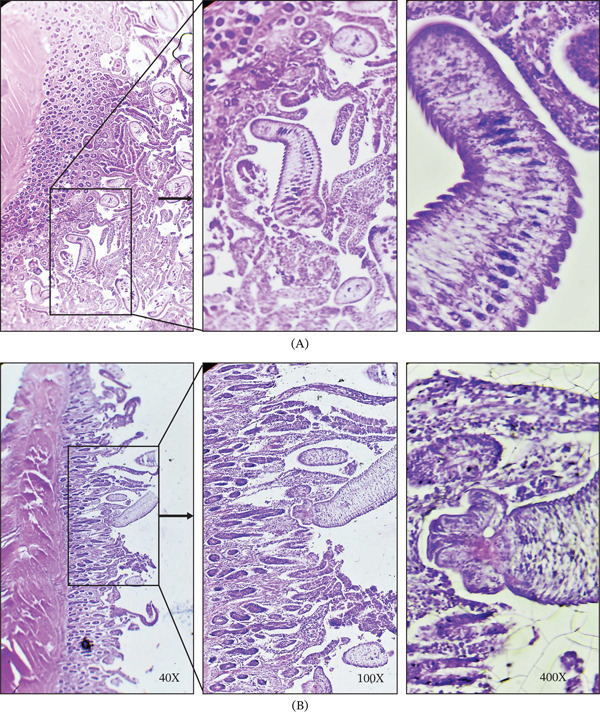
Histopathological slide of the intestine of backyard chicken. (A) Severe infestation of *A. sphenoides* and its segments embedded in the intestinal villi and the destruction of lamina propria. (B) Severe infestation of *R. cesticillus* and its segments embedded in the intestinal villi and the destruction of lamina propria. H&E staining *(*40x, 100x, and 400x).

**Figure 6:**
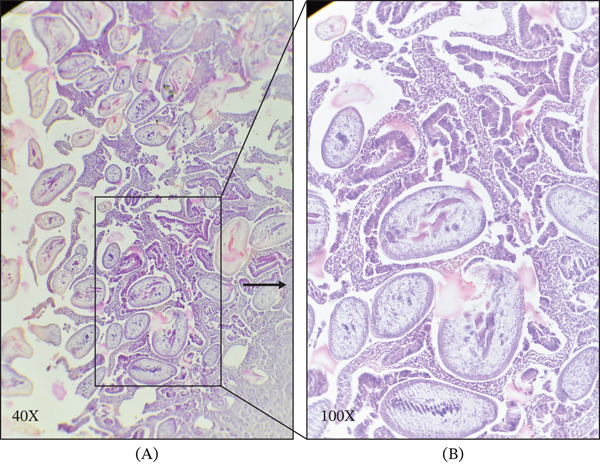
Cestodiasis causes necrotizing and invades the (A) intestinal epithelium and (B) segments and eggs of cestodes. H&E staining (40x, 100x, and 400x).

Histopathological examination revealed *H. gallinarum* in the submucosal and serosal layers of the intestinal wall (Figure 7). Nematode invasion resulted in coalescence of intestinal glands, which appeared as disruption of cellular masses within the lamina propria. Nematode‐associated hemorrhagic enteritis was characterized by severe loss of microvilli and degenerative changes throughout the intestinal mucosa. Due to *H. gallinarum* infestation, the muscularis mucosa and digestive glands are disrupted. The chickens with mixed infection of *H. gallinarum* and *A. galli* showed the inflammatory response, involving the full thickness of the gastrointestinal wall, and notable muscular and structural disruptions.

**Figure 7:**
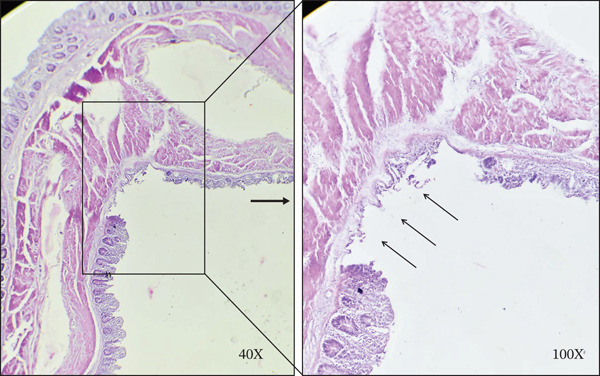
Histopathological slide of naturally infected chicken intestine with H&E staining demonstrating intestinal glands, basement membrane degradation, and lamina propria. Magnification 40x and 400x.

## 4. Discussion

The present study focused on parasitic infections in backyard poultry birds, which are affected by helminths, specifically cestodes and nematodes. Furthermore, backyard chickens were exposed to various arthropods, which serve as intermediate hosts of many parasites. In addition, their feeding practices increase their vulnerability to parasitic infections. Previous studies supported the premise that feeding habits are directly proportional to parasitic spread [25, 33].

Findings of the present study revealed that 89.47% of domestic chickens were infected with cestodes. These findings were close to those reported by [34], who found 64.30% in Nigeria; moreover, [35] reported 75.8% in Quetta, Pakistan. Although [36] and Yousfi et al. [37] stated higher infection rates in Eritrea (80.0%) and in Algeria (95.67%), [38] reported that five cestode species were found with a low individual chicken prevalence (*Raillietina tetragona*, 4.7%; *R. echinobothrida*, 3.2%; *R. cesticillus*, 5.2%; *C. infundibulum*, 4.4%; and *Hymenolepis cantaniana*, 4.4%).

The present study revealed four cestode species in chickens, with the highest prevalence of 32.89% for *C. diagonopora*. This result is close to the findings of [39], who reported 31.66% prevalence. Furthermore, [40] found a high prevalence of 94.5%. In addition, [41] have recorded slightly lower prevalence, that is, 24.1%, and others have reported a prevalence of less than 5% [42–43].

Followed by the second species, *C. infundibulum*, which has 27.63% prevalence, these records are closest to [44], which reported 13.0%, and [40], which reported 89.5%. Conversely, [26, 42, 45, 46] reported a prevalence of less than 10.0%.

The prevalence percentage of the third cestode species *R. cesticillus* recorded in chickens during the present study was 14.47%. These results are close to those reported in [47] (12% in Morocco) and [48] (12.37% in the Al‐Diwaniya region). In comparison, [49] reported a 12% prevalence, [50] reported a 3.33% prevalence. [51] mentioned 18.14% prevalence in district Mardan, KPK, Pakistan. Furthermore, other studies report a prevalence within a similar range for the same parasite [25, 34, 44, 50–52].

The prevalence of the fourth species, *A. sphenoides* scolex cestode, was 3.94%. Though *A. sphenoides* scolex is primarily known as a tapeworm species that infects domestic birds, there is limited specific information available in the existing literature about *A. sphenoides* in chickens; it is important to note that chickens can be susceptible to various types of tapeworm infections. Recently, *A. sphenoides* was reported in free‐ranging poultry in the Kashmir Valley, India [53]. This is the first report of this parasite in backyard poultry in Sindh, Pakistan. The infection of *A. sphenoides* in backyard chicken might be due to the availability of the intermediate host (earthworm) [54].

In addition, the prevalence of Ascaridia was 35.53%, which is comparable to 35.35% and 22.6% in Kashmir and Shimoga, India [55, 56], 45.6% in Bangladesh [16]. Moreover, reports from northern Jordan show Ascaridia galli infection female 28%, male 43%[57], and reports by others [14, 27].

Backyard chickens hunt for food in the ground, which is contaminated with insects and mollusks that act as intermediate hosts for parasitic infective stages [58]. *H. gallinarum* is very common, and this may be due to changes in the agroecosystem. Starting with a small infection, *H. gallinarum* can cause severe disease. However, it also transmits the protozoan that causes histomoniasis [59]. *A*. *galli*, on the other hand, inhibits development and production while causing mucosal irritation and inflammation, which affects how food is absorbed [60].

The prevalence of *H. gallinarum* observed throughout the study was 43.42%, whereas another study reports *H. gallinarum* to be 72.5% in Makurdi metropolis [61]. However, the prevalence of *H. gallinarum* in backyard chickens reported by many researchers is lower than that observed in the present study. Examples include 24.79% in Nigeria [62], 32.6% in central Ethiopia [26], 30.2% in Mexican tropics [63], 24.0% in Jammu, India [14], 24.0% in Golestan Province, north of Iran [64], and 20.0% in District Hyderabad, Pakistan [27, 40].

Accordingly, the prevalence of helminth infections varied across areas and countries and can be primarily attributed to management and environmental factors. The fact that the cited studies were conducted at various times of the year has affected the interpretations. Variations in prevalence rates could result from differences in animal age, intermediate hosts, individual host resistance, and environmental factors. Present findings in comparison with other studies conclude that a higher prevalence of helminths is anticipated in chickens reared in free‐range or backyard environments [65]. It can be stated that both nematodes can complete their life cycle without intermediate hosts [66].

Additionally, our findings are in complete agreement with [55], who observed a correlation between the incidence of helminth infections and the average worm burden with declining temperatures and precipitation, with helminth infection incidence peaking in September and troughing in December and January. During the warm, rainy months, a heavy infection may be brought on by high mean temperatures and high relative humidity, as they reduce birds′ resistance to infection and favor infection. Hence, the reduced infection incidence during the winter may be related to cold weather, which may also aid in halting parasite growth in the host and its surroundings.

Necropsy findings showed the chickens were anemic, and enteritis, diarrhea, and hemorrhages were commonly seen in heavily infested chickens. Cellular infiltration, enteritis, atrophied villi, and formation of granulomas and other lesions were reported by [67] and [68]. In another study, hemorrhages, ulceration, nodular growth, and battered mucosal epithelium of the intestine were found in poultry cestodiasis [68]. These findings are like or at least consistent with the current study. Histopathological findings revealed the degeneration of intestinal villi and erosion of mucosal epithelium in all infected birds. Cestodiasis causes necrosis and invades the intestinal epithelium, shedding eggs throughout the intestinal layer. A large inflammatory response in the intestinal lumen and severe muscular abnormalities were seen in the GIT, including hyperemia, hemorrhage, inflammatory cell infiltration, and epithelial erosion. In previous studies, less growth of the intestine and degeneration of villi were observed in cestode‐infected fowl as compared to uninfected birds [69].

*R. cesticillus* and *A. sphenoides* invasion was found in the intestinal epithelial layer. Histopathological study revealed that the scolexes of *R. cesticillus* and *A. sphenoides* were deeply burrowed into the mucosa and the infection site. [69] reported the same findings about *A. sphenoides* in free‐range chicken.

## 5. Conclusions

The present baseline study established that free‐range backyard chickens in the rural areas of Tandojam are heavily infested with mixed helminth parasites and highlights the scale of the parasitic disease problem in free‐range chickens. Four species were identified: *C. digonopora*, *C. infundibulum*, *R. cesticillus*, and *A. sphenoides*, with *C. digonopora* recording the highest prevalence. The discovery of *A. sphenoides* in backyard chickens is the first confirmed case of this species in the Sindh Province, Pakistan, in this regional epidemiological significance. Two nematode species were also identified, *H. gallinarum* and *A. galli*, both of which showed marked histopathological lesions, including inflammatory cellular infiltration, villous degeneration, and mucosal erosion. Altogether, these significant findings will need to be urgently incorporated into parasite control and surveillance programs for backyard poultry across Sindh province. Because of weak biosecurity measures and unrestricted access to intermediate hosts, backyard chickens will remain highly infested with helminths.

## 6. Recommendations

Based on the results of the present study, a routine and seasonal anthelmintic treatment program for backyard chickens should be established. Target the cestode and nematode species identified in this study to effectively reduce parasitic infestation. Farmers should be educated on basic biosecurity procedures, including proper litter management, sanitation, and reducing environmental conditions that favor intermediate host populations. Future research should include uninfected control groups and an induced group, expand sample sizes, and apply inferential statistical analyses to more accurately assess the pathological effects of helminth infections. The regional novelty of *Amoebotaenia sphenoides* warrants dedicated follow‐up investigation into its intermediate host biology, geographical distribution, and pathogenic potential in backyard poultry populations across Sindh, Pakistan.

## Author Contributions

Sahar Bhutto and Sadaf Khushk equally contributed to this research.

## Funding

No funding was received for this manuscript

## Conflicts of Interest

The authors declare no conflicts of interest.

## Data Availability

The data that support the findings of this study are available on request from the corresponding author. The data are not publicly available because of privacy or ethical restrictions.
